# The Impact of Information Technology on Radiology Services: An Overview: Article based on PhD dissertation to obtain the degree of doctor in medical sciences, defended at the University of Antwerp on July 14, 2016

**DOI:** 10.5334/jbr-btr.1184

**Published:** 2016-11-19

**Authors:** Erik R. Ranschaert

**Affiliations:** 1 University Hospitals of Leuven, Dpt. of Radiology, Leuven, Belgium

**Keywords:** Digital imaging, Imaging informatics, Information technology, Radiology, Social media, Teleradiology

## Abstract

The main objective of this paper is to provide an overview of the impact of information technology on radiology services during the past 15 years and to promote awareness of the digital revolution that is taking place in health care, including radiology. The combination of two major innovations is playing a central role in this revolution, namely, the Internet and the digitisation of medical information. The various stages of the Internet development and their relationship with the almost simultaneously ongoing digitisation of the radiology department are described. The onset of teleradiology services and the more recent trend toward the usage of cloud-based networks and services are explained. The recent changes in digital communication and electronic transmission of medical information are discussed, hereby paying attention to the value of social media in medicine and radiology in particular. Finally, the future prospects of health care and medical imaging are outlined in the spotlight of today’s major trends, and the role of the radiologist in this quickly changing environment is redefined.

## Introduction

During the past few decades, the limits of imaging informatics have been pushed beyond traditional borders due to several major changes in computer and communication technology. Through the introduction of new technologies, such as the World Wide Web, wireless connectivity, and, more recently, the social networks, significant progress has been made in the way radiological services can be delivered. The Internet has become an indispensable gateway for electronic transmission and sharing of health-related data, a process known as “e-Health”. Many different types of e-Health are currently becoming available. In many hospitals, the electronic health record (EHR) is being introduced, which allows a longitudinal and complete electronic record of the patient’s health information [2,3]. This EHR should not only automate and streamline the physician’s workflow but also allow patients to gain control over their health data through online portals.

Besides this, a growing number of electronic devices and sensors are becoming connected to the Internet and gradually shaping the Internet of Things (IoT). This IoT enables real-time collection of an enormous amount of autonomous, health-related data, even on a global scale. Those wireless “smart wearables” can be used for a wide variety of health-related purposes, such as monitoring of heart rate, body temperature, mobility, sugar levels, and the like [4]. Not only the volume of electronically shared health data is increasing exponentially, but also the power for processing these data. By cross-linking information obtained from intelligent cloud-based data analysis with enormous amounts of genetic data, it has become possible to generate information that is useful for providing personalised care.

All this is resulting in numerous opportunities for telemedicine, an evolution that should be advantageous for both health care professionals and patients. There is also great confidence in the potential of telemedicine to reduce the public expenditure on health care. The European eHealth Action Plan 2012–2020 aims to address and remove the remaining barriers with the intention to benefit fully from an interoperable eHealth system in Europe. The recommendations as published by the EU eHealth Task Force for reorganising health care in Europe are mainly based on the use of information technology. According to these recommendations, the patient should be the owner and controller of his or her health data, the governments should create legal solutions to “liberate” health data, and health institutions should leave their “silo mentality” to facilitate the sharing of health data [5].

## Historical Overview

The Internet went through different stages, going from the early “static” Web 1.0 to the current “semantic” Web 3.0. Almost simultaneously with these developments, the digitisation process in radiology made progress. The combination of increased Internet accessibility with the digital radiology images availability has resulted in new methods for storing and distributing medical images. In the Web 1.0 phase most attention went to technical issues, such as the preservation of image quality and the accessibility to high-bandwidth connections for easy transmission of large and “heavy” image series. In the Web 2.0 or “social networking” phase, most attention shifted to issues related to the quality of services and the protection of patient’s privacy took place, mainly in the context of teleradiology services. Currently, the “semantic” Web 3.0 is on the march, through which large amounts of information (big data) can be shared and processed intelligently, with unprecedented speed and power. At the basis of this evolution is the emergence of cloud computing. Gradually, more cloud-based services are being offered for radiological purposes, such as online archiving, sharing, and post-processing of radiological images.

The European Forum for Radiologists (EUFORA) mailing list was one of the first radiology applications that were developed shortly after the launch of the public Internet. The mailing list was connected to a web-based image database (CONRAD). After a case was submitted by a member of the mailing list, an e-mail was automatically sent to all the other list members, with the invitation to provide their comments. The images could be viewed immediately by using the included hyperlink. In that period, several other similar Internet services were created, with the main intention to facilitate communication between radiologists on an international level, both for professional and educational purposes. These projects were the basis for web-based image distribution. A more detailed overview of these early developments is provided in chapters 3 and 4 of E. Ranschaert’s thesis [1].

The switch from an analogue to a digital working environment put the radiologists at the front line of producing and distributing digital images. New dedicated software products were developed, such as PACS, RIS, and HIS. This also led to the development of standards for the electronic transmission of radiological images, such as the DICOM standard [6]. The digitisation of radiology and the fast-growing availability of high-bandwidth Internet were the main contributors to the onset of teleradiology, the transmission and reading of radiological images at distance, often from outside the hospital. In many radiology departments, teleradiology services became part of the daily workflow, mainly to solve such problems as increasing workloads, shortages in radiological capacity, and demand for subspecialty advice.

The development of teleradiology services followed a different course in the US compared to the EU, mainly because of the differences in markets and health care systems. Within a few years, a very competitive and even predatory teleradiology market was created the US. In the EU, however, a more fragmented implementation of teleradiology services took place, not only due to the differences in national health care systems but also because of cultural and linguistic differences, and a rather heterogeneous spread of PACS throughout Europe.

The increasing prevalence of teleradiology generated many in-depth discussions, however, mainly about issues related to financing, quality management, patient safety and security, and professional certification for cross-border transactions. For these reasons, several radiological societies, such as the European Society of Radiology (ESR) and American College of Radiology (ACR) published their teleradiology guidelines or “White Paper”. Despite these struggles, teleradiology did emerge as the largest and most mature segment of the overall telemedicine industry. More information about this topic can be found in chapter 5 of E. Ranschaert’s work [1].

## Disruptive Innovations

The more recent popularity of mobile devices combined with the exponentially growing availability of mobile applications has a significant impact on the fast development of new e-Health services. The increasing availability of mobile computing hardware and software is particularly relevant to radiology, where the day-to-day workflow is intimately intertwined with digital tools. What started in the early days of the Internet with websites, mailing lists, and newsgroups has evolved into a digital society where patients can and want to share their health information with almost whomever they want using social media and other applications. These “disruptive” innovations are progressively replacing earlier established communication tools.

An increasing number of health care professionals are also using social media for professional purposes, although most public platforms are not specifically developed and secured for such a purpose. In the recently conducted RANSOM survey, it was demonstrated that 85 percent of radiologists intensively use social media, mostly for a mixture of private and professional reasons [1,7]. The professional use of social media could also be considered as a tool to optimise the perceived value and visibility of the radiologist. This idea is congruent with the ACR “Imaging 3.0” initiative, for example, in which radiologists are being called upon to make a more visible, active role in health care and to call attention to the work they do and its value to patients. Radiologists connecting with patients through social media could enable them to provide general information about radiology and to gain valuable insight in patients’ perceptions about radiological examinations and services [1,7]. It has been shown too that radiologists are using social media such as WhatsApp to discuss medical images with colleagues (e.g., for obtaining a second opinion).

**Figure 1:**
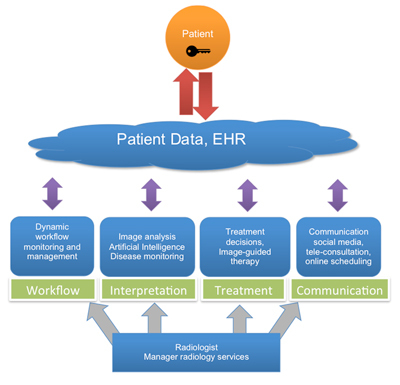
The radiologist as manager can use information technology (IT) in four major fields: namely, the management of workflow, the interpretation of images, the treatment decision-making, and the communication with clinicians and patients. Full interoperability between health data resources and unlimited exchange of and unrestricted access to these data is hereby essential, independent from place and time, and with full approval of the patient.

Although using such public platforms is not compliant with the existing European legal framework regarding the protection of patient privacy, the ability to obtain an expert opinion from a colleague within very short notice might be of benefit for the patient, especially in a life-threatening situation [8]. Radiologists should therefore be encouraged to use dedicated social media that follow the legal requirements and can optimally safeguard patient privacy. Several such platforms are already available in several countries, such as “Siilo”, “MDLinking”, “Kanta Messenger”, “Sermo”, and “Figure 1”. For more detailed information about the usage of social media by radiologists and patients we refer to chapter 6 of E. Ranschaert’s work [1].

This increasing user-based demand for access to digital data is causing a gradual degradation of the traditional health care model, in which the hospital plays a central role. The walls surrounding the traditional hospital-based information-silos are progressively being “deconstructed”, which causes a shift from the classic hospital-centric model towards a more patient-centric model of care. In this model, all relevant patient data are shared fluently between all stakeholders of the health care process. The information stream needs to become more “liquid” so that it can run as easy as water in a river. This change will guide us further to the construction of the “liquid hospital”. In this future scenario, operational decisions will heavily rely on real-time data analysis of individual medical measurements and outcomes. All relevant information will be available for any health care provider, independent of time and place. The first evidence of this ongoing shift is visible in the progressive introduction of patient portals [3].

Internet portals can provide access to different services, such as online appointment scheduling, video consultations, monitoring of vital functions, the provision of second opinions, and access to medical results, including radiological images and reports. Such innovations are paving the way to greater patient empowerment, meaning that patients can be actively involved in the management of their health process, which will also transform the way radiological services are provided.

Further evidence of the ongoing shift towards the patient-centric model can be found in the changes that are being made by Melvin Samson at the new Karolinska hospital (Nya Karolinska Solna) in Stockholm [9]. The entire infrastructure and organizational model of the Swedish hospital is adapted to the “Patienten först” principle. Four hundred *patient flows* are being created, which are organized around different themes (e.g., breast cancer). *All* specialties involved in the process of breast cancer, such as oncology, surgery, and radiology, are grouped around the same flow, which eliminates the necessity for the patient to move from one department to the other. In this model, such traditionally fully separated departments as surgery and internal medicine will disappear. The main intentions are to deliver optimal personalised treatments to patients and to reach a higher cost-effectiveness in providing care.

## Redefining Radiology

As explained in the previous section, patients are increasingly becoming empowered to actively monitor and manage their health, and health care providers on their side are developing new services to facilitate patients in this process. An even greater enhancement to care can be expected through the provision of a personalised electronic key to patients, allowing them to grant access to their data to a health care provider of choice [10]. Due to the increasing ability to collect data from millions of wearable devices and the possibility to mine these data with cloud-based techniques, the concept of personalised medicine is progressively taking a leading position. Medical interventions and medications will become tailored to individual patients based on their predicted responses to the disease. It will also be possible to make such decisions based on the combination of morphologic information from medical images with genomic data, a technology known as “radiogenomics”. The term “radiomics” refers more to the automated morphologic analysis of radiological images with new cloud-based deep-learning techniques that convert these images to mineable data. The term “radiogenomics” is preferably used for the process of correlating the data obtained from radiomics with genomic (genetic) information of a disease or patient.

It can be predicted that all these changes will move radiology from a mainly diagnostic to a more treatment-related type of imaging specialty [1]. Besides the morphologic evaluation of disease over time, functional measurements will become indispensable to grade a disease and monitor treatment (responders vs. non-responders). Additional proof of this statement can be found in the development of such new image-guided treatment techniques as MRI high-intensity focused ultrasound (MRI-HIFU) and image-guided radiation therapy (IGRT). By using image-guided radiation therapy (IGRT), repeated imaging can be performed during the treatment to identify changes in the tumour’s size and location, allowing adjustment of the patient’s position or the radiation dose. This can increase the accuracy of radiation treatment and may allow reductions in the planned volume of tissue to be treated, thereby decreasing the total radiation dose to normal tissue.

At the VUmc Cancer Center Amsterdam, the Netherlands, the first radiation therapy machine in Europe with an integrated MRI scanner was recently installed. The greatest advantage in using MRI during radiation therapy lies in the fact that the implantation of gold particles for marking the tumour becomes unnecessary, since with MRI it’s possible to discern healthy soft tissue from tumour more accurately than with plain CT. This other example of image-based treatment indicates that a more active engagement of the radiologist in the treatment process will be required [11]. In addition, much research is being conducted in molecular imaging to develop “probes” or biomarkers, which will be used to image particular targets or pathways related to specific diseases and treatments.

The ability to image fine molecular changes opens a great number of exciting possibilities, not only for the detection but also for the treatment of disease. It is expected that these and other innovative image-guided techniques will allow more precise and less invasive interventions, which can give a boost to radiology, under the condition that an environment is created in which radiologists and other specialists are able to collaborate more intensively. In the perspective of such upcoming changes, radiologists will have to engage actively in making clinical and therapeutic decisions in a multidisciplinary environment, and they will have to be able to go along with non-invasive image-guided treatments (Figure 1). The “new” radiologist can also play a crucial role in efficiently communicating all relevant information to both clinicians and patients to assist them in making the optimal decisions regarding diagnosis and treatment.

Another milestone in IT is the development of artificial intelligence (AI), which made a huge step forward in recent years. IBM is developing the highly intelligent software with the code name “Avicenna”, which is based on the IBM-Watson computer system. The company plans to leverage the Watson Health Cloud “to analyse and cross-reference medical images against a deep trove of lab results, electronic health records, genomic tests, clinical studies and other health-related data sources” [12]. Many deep-learning algorithms still need to be developed, tested, and approved, however, before it will be possible to implement AI routinely for clinical purposes. It can be expected that in 5 to 10 years from now, Avicenna or a similar AI system will be sufficiently trained to act as a first filter for analysing all sorts of medical images that are later examined by doctors. This does not mean that those systems will be able to completely “replace” radiologists. Such supercomputers will probably act as a provider of second opinions, helping to confirm a radiologist’s suspicion of an unusual or difficult diagnosis. This in turn could cut down on superfluous and unnecessary testing, which saves time for the patient, eliminates unnecessary radiological exposure, and reduces costs [13]. In addition, Watson could also play a particularly crucial role in serving remote areas with insufficient medical care. The availability of wireless access to the cloud for mobile devices will undoubtedly contribute to this positive evolution.

It should be questioned, however, to what extent in the future image analysis will be performed by computers instead of radiologists and what effect this will have on the “ownership” of the technology. Such evolution could possibly translate into a challenge about the value of the work and the financial compensation of radiologists [14]. It’s the authors’ opinion that radiologists should start with embracing AI as soon as they can, with the main intention to participate in AI research with the objective of creating IT tools that can add value to radiology services. Many radiologists are confronted with a high workload, which is not only caused by the growing demand in medical imaging but also by the increasing complexity of radiological examinations (e.g., integration of nuclear imaging with radiology). The demand to integrate EHR information in the radiological report is probably another contributing factor. Radiologists should try to use AI for managing their workloads more efficiently. AI could be used to do preliminary reads of imaging studies, for example, so that radiologists are able to use Watson’s information to make their final reports. By doing so, radiological error rates could possibly be reduced. In this context, AI should rather be regarded as a form of intelligence amplification (IA) for radiologists, a technique enabling them to add value to the radiology report [11]. In other words, AI could be used to consolidate the radiologists’ role instead of replacing them.

To summarise, the radiologist in his role as manager could make optimal use of information technology (IT) in four principal fields: namely, the management of workflow, the interpretation of images, the treatment of patients, and the communication with clinicians and patients (Figure 1). Being confronted with an increasing workload IT can be used to monitor the work processes, to optimise the workflow, and to simplify procedures. The integration of automated imaging findings and quantitative “omics” data with relevant her information could optimise the information provided in the radiology report. In this data-rich environment, in which the context is king, radiologists should embrace AI to simplify the process of enriching their reports with these data. Use of IT should also enable radiologists to assist in selecting more personalized or less-invasive treatment options for the patient. This more holistic approach to the patient’s treatment can be established in multidisciplinary meetings or teams centred on a specific disease or body part.

**Figure 2:**
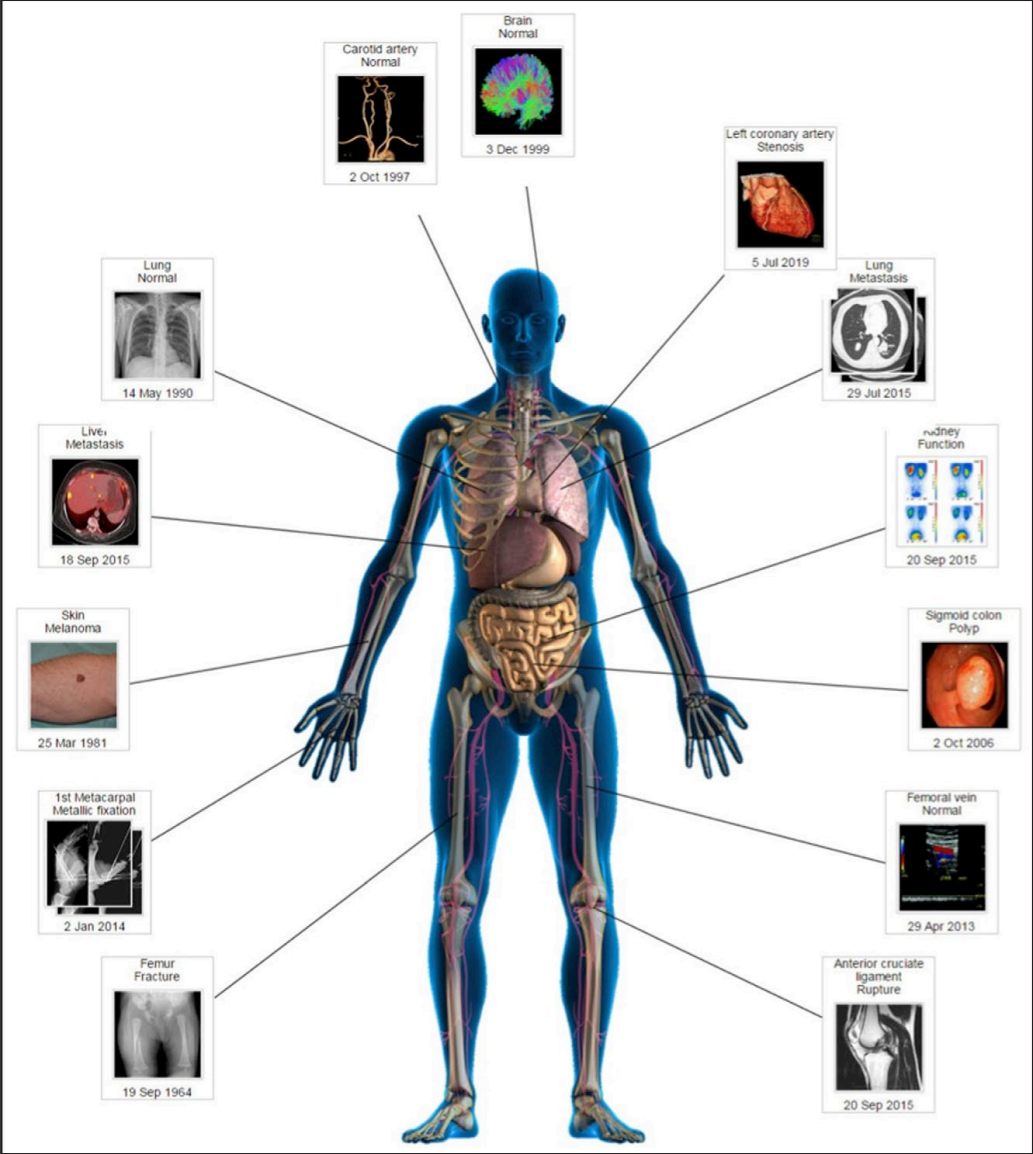
A software application to make structured multimedia reports, consisting of a graphic presentation of the patient, shows interactive key images of each anatomic region. By clicking on these images, all relevant information of the related body part becomes visible. (Used with the permission of David J. Vining).

Finally, IT could support the communication of radiologists with clinicians and patients and facilitate a more direct communication between radiologists and patients. Enabling patients to contact the radiologist for an explanation of the findings could create a greater awareness of the crucial role of radiologists. With portals providing access to radiology reports, this will become a requirement. The use of “multimedia reports” could also be considered, in which the information is displayed in a simplified but more structured and interactive manner (Figure 2). Crucial in such a management model is the optimal interoperability between health data systems and the 24/7 availability of all relevant patient data. The patient should be the full owner of these data and therefore should have the possibility to decide who is authorized to make use of them. It will also be necessary for health care organisations and policymakers to fundamentally change their vision and decision-making related to key areas in this field. They will have to support the seamless sharing of data with appropriate security and privacy protections and the creation of new policy guidelines for national and international collaboration. This way, it should be possible to reach the destination of the patient-centric health care model.

## Conclusion

An unstoppable revolution is taking place which is leading to a significant change in the position of medical imaging. The role of the radiologist should be redefined as that of the manager and service provider that is making optimal use of information technology to align his or her operations and workflow to a modifying environment in which radiology is more actively involved in the process of diagnosis, treatment, and communication. A better awareness of this on-going metamorphosis in radiology should help radiologists to capitalise on the new possibilities that are created and to improve their services for the benefit of the patient.

## Addendum

More information on the thesis of E. Ranschaert, with the title *The Impact of Information Technology on Radiology Services*, can be found at the following:

- Slides of the dissertation can be found here: http://bit.ly/2a2m5cn .
- Download the dissertation here: http://bit.ly/2a5jAUo .
